# Supplementary material for: Power analyses to inform clutch sampling design to determine the breeding sex ratio in populations with multiple paternity
Source: PeerJ. 2025 Oct 28;13:e20165. doi: 10.7717/peerj.20165 (PMC12577575; doi:10.7717/peerj.20165)
Supplement: Supplemental Information 1 — We collected these data via nightly nesting beach patrols from end of December through June of each nesting season. We identified nesting females through existing metal flipper tags or we designated new females and tagged them with metal flipper tags. Several days after nests hatched (or 60 days after the lay date, if hatching was not observed), we excavated all nests that could be found, and we calculated the total number of eggs as the sum of eggshell fragments (only counting eggshell fragments that were at least half an egg), dead and live hatchlings, and unhatched eggs. Data collection for the 2020 season was interrupted due to the COVID-19 pandemic, so we did not record any data past April 2nd. We calculated the distribution of the number of clutches per female based on data from the 2021–2023 nesting seasons, excluding clutches missing identified females but including clutches missing egg counts (Table 1). We calculated the distribution of the number of eggs per clutch based on data from all nesting seasons, excluding nests missing egg counts but including nests missing identified females and lay dates (Table 1). Distributions visually did not vary much between nesting seasons and were approximately normally distributed. All field work and data collection, including animal capture and tagging, was performed following protocols approved by the Institutional Animal Care and Use Committee at Florida State University (permits 1803, and PROTO202000076) and the Brazilian Ministry of Environment (MMA), Chico Mendes Biodiversity Conservation Institute (ICMBio), and Biodiversity Authorization and Information System (SISBIO), approval #69389-12. [file peerj-13-20165-s001.docx]

| **Season** | **Lay Date** | **Female** | **Eggs Total** |
| --- | --- | --- | --- |
| 2020 | NA | NA | 75 |
| 2020 | NA | NA | 185 |
| 2020 | NA | NA | 84 |
| 2020 | 19-12-14 | NA | 86 |
| 2020 | 19-12-15 | NA | 85 |
| 2020 | 19-12-19 | NA | 168 |
| 2020 | 19-12-26 | 2 | 79 |
| 2020 | 19-12-27 | 3 | 101 |
| 2020 | 19-12-28 | 4 | 97 |
| 2020 | 19-12-31 | NA | 163 |
| 2020 | 20-01-03 | 5 | 87 |
| 2020 | 20-01-06 | 2 | 113 |
| 2020 | 20-01-08 | 6 | 145 |
| 2020 | 20-01-08 | 4 | 99 |
| 2020 | 20-01-08 | 3 | 78 |
| 2020 | 20-01-13 | 7 | 90 |
| 2020 | 20-01-13 | 8 | 107 |
| 2020 | 20-01-13 | NA | NA |
| 2020 | 20-01-15 | 9 | 87 |
| 2020 | 20-01-16 | 10 | 97 |
| 2020 | 20-01-16 | 11 | 123 |
| 2020 | 20-01-16 | 5 | 84 |
| 2020 | 20-01-17 | 2 | 113 |
| 2020 | 20-01-17 | 12 | 102 |
| 2020 | 20-01-18 | 6 | 132 |
| 2020 | 20-01-19 | 4 | 128 |
| 2020 | 20-01-20 | 3 | 65 |
| 2020 | 20-01-22 | 13 | 101 |
| 2020 | 20-01-24 | 1 | 151 |
| 2020 | 20-01-24 | 8 | 113 |
| 2020 | 20-01-25 | 7 | 93 |
| 2020 | 20-01-25 | 14 | 92 |
| 2020 | 20-01-27 | 9 | 99 |
| 2020 | 20-01-27 | 11 | 116 |
| 2020 | 20-01-29 | 2 | 92 |
| 2020 | 20-01-29 | 10 | 83 |
| 2020 | 20-01-29 | 6 | 133 |
| 2020 | 20-01-30 | 4 | 120 |
| 2020 | 20-01-30 | 12 | 98 |
| 2020 | 20-01-31 | 5 | 72 |
| 2020 | 20-02-01 | 15 | 112 |
| 2020 | 20-02-02 | 16 | 72 |
| 2020 | 20-02-03 | 8 | 93 |
| 2020 | 20-02-04 | 1 | NA |
| 2020 | 20-02-05 | 7 | NA |
| 2020 | 20-02-05 | 17 | NA |
| 2020 | 20-02-06 | 13 | NA |
| 2020 | 20-02-07 | 14 | 94 |
| 2020 | 20-02-08 | 9 | NA |
| 2020 | 20-02-08 | 18 | NA |
| 2020 | 20-02-09 | 4 | NA |
| 2020 | 20-02-09 | 19 | NA |
| 2020 | 20-02-09 | 11 | NA |
| 2020 | 20-02-09 | 12 | NA |
| 2020 | 20-02-09 | 20 | NA |
| 2020 | 20-02-10 | 6 | 150 |
| 2020 | 20-02-10 | 21 | NA |
| 2020 | 20-02-11 | 10 | 87 |
| 2020 | 20-02-11 | 15 | 86 |
| 2020 | 20-02-12 | 16 | NA |
| 2020 | 20-02-13 | 22 | 54 |
| 2020 | 20-02-13 | 5 | 87 |
| 2020 | 20-02-13 | 8 | NA |
| 2020 | 20-02-15 | 23 | 66 |
| 2020 | 20-02-15 | 1 | NA |
| 2020 | 20-02-17 | 7 | NA |
| 2020 | 20-02-17 | 13 | 106 |
| 2020 | 20-02-17 | 24 | 99 |
| 2020 | 20-02-17 | 17 | NA |
| 2020 | 20-02-18 | 14 | 104 |
| 2020 | 20-02-19 | 6 | NA |
| 2020 | 20-02-19 | 4 | 115 |
| 2020 | 20-02-19 | 25 | NA |
| 2020 | 20-02-19 | 11 | NA |
| 2020 | 20-02-19 | 12 | NA |
| 2020 | 20-02-19 | 19 | 107 |
| 2020 | 20-02-19 | 9 | NA |
| 2020 | 20-02-20 | 26 | 75 |
| 2020 | 20-02-20 | 20 | 124 |
| 2020 | 20-02-20 | 21 | NA |
| 2020 | 20-02-21 | 27 | NA |
| 2020 | 20-02-21 | 28 | NA |
| 2020 | 20-02-21 | 15 | NA |
| 2020 | 20-02-23 | 29 | NA |
| 2020 | 20-02-23 | 10 | NA |
| 2020 | 20-02-23 | 8 | NA |
| 2020 | 20-02-23 | 16 | NA |
| 2020 | 20-02-24 | 30 | NA |
| 2020 | 20-02-25 | 31 | NA |
| 2020 | 20-02-26 | 1 | NA |
| 2020 | 20-02-27 | 13 | NA |
| 2020 | 20-02-27 | 22 | NA |
| 2020 | 20-02-28 | 23 | NA |
| 2020 | 20-02-28 | 24 | NA |
| 2020 | 20-02-28 | 7 | NA |
| 2020 | 20-02-28 | 17 | NA |
| 2020 | 20-02-28 | 6 | NA |
| 2020 | 20-02-28 | 4 | NA |
| 2020 | 20-02-28 | 14 | NA |
| 2020 | 20-02-29 | 12 | NA |
| 2020 | 20-02-29 | 11 | NA |
| 2020 | 20-02-29 | 18 | NA |
| 2020 | 20-02-29 | 19 | NA |
| 2020 | 20-02-29 | 32 | NA |
| 2020 | 20-03-01 | 9 | NA |
| 2020 | 20-03-01 | 20 | NA |
| 2020 | 20-03-02 | 21 | NA |
| 2020 | 20-03-03 | 15 | NA |
| 2020 | 20-03-03 | 8 | NA |
| 2020 | 20-03-03 | 33 | NA |
| 2020 | 20-03-03 | 28 | NA |
| 2020 | 20-03-03 | 25 | NA |
| 2020 | 20-03-04 | 34 | NA |
| 2020 | 20-03-04 | 16 | NA |
| 2020 | 20-03-06 | 27 | NA |
| 2020 | 20-03-06 | 10 | NA |
| 2020 | 20-03-06 | 26 | NA |
| 2020 | 20-03-06 | 29 | NA |
| 2020 | 20-03-08 | 1 | NA |
| 2020 | 20-03-08 | 6 | 138 |
| 2020 | 20-03-08 | 13 | NA |
| 2020 | 20-03-08 | 35 | NA |
| 2020 | 20-03-08 | 36 | 83 |
| 2020 | 20-03-09 | 31 | NA |
| 2020 | 20-03-10 | 23 | NA |
| 2020 | 20-03-10 | 17 | 94 |
| 2020 | 20-03-10 | 11 | 127 |
| 2020 | 20-03-10 | 37 | NA |
| 2020 | 20-03-10 | 22 | 90 |
| 2020 | 20-03-10 | 14 | NA |
| 2020 | 20-03-11 | 19 | NA |
| 2020 | 20-03-11 | 24 | NA |
| 2020 | 20-03-11 | 9 | 100 |
| 2020 | 20-03-11 | 12 | 101 |
| 2020 | 20-03-11 | 20 | 61 |
| 2020 | 20-03-12 | 7 | NA |
| 2020 | 20-03-12 | 38 | NA |
| 2020 | 20-03-12 | 21 | NA |
| 2020 | 20-03-12 | 39 | NA |
| 2020 | 20-03-13 | 33 | NA |
| 2020 | 20-03-13 | 40 | NA |
| 2020 | 20-03-13 | 41 | NA |
| 2020 | 20-03-14 | 42 | NA |
| 2020 | 20-03-14 | 8 | NA |
| 2020 | 20-03-14 | 16 | NA |
| 2020 | 20-03-15 | 28 | NA |
| 2020 | 20-03-15 | 25 | NA |
| 2020 | 20-03-16 | 34 | NA |
| 2020 | 20-03-17 | 26 | 28 |
| 2020 | 20-03-17 | 29 | NA |
| 2020 | 20-03-18 | 30 | NA |
| 2020 | 20-03-18 | 1 | NA |
| 2020 | 20-03-19 | 27 | NA |
| 2020 | 20-03-20 | 35 | 100 |
| 2020 | 20-03-20 | 36 | 96 |
| 2020 | 20-03-20 | 31 | NA |
| 2020 | 20-03-20 | 37 | NA |
| 2020 | 20-03-20 | 19 | NA |
| 2020 | 20-03-20 | 22 | NA |
| 2020 | 20-03-21 | 12 | NA |
| 2020 | 20-03-21 | 23 | NA |
| 2020 | 20-03-21 | 14 | NA |
| 2020 | 20-03-21 | 43 | NA |
| 2020 | 20-03-21 | 11 | NA |
| 2020 | 20-03-21 | 20 | NA |
| 2020 | 20-03-22 | 9 | NA |
| 2020 | 20-03-22 | 21 | NA |
| 2020 | 20-03-23 | 44 | NA |
| 2020 | 20-03-23 | 33 | NA |
| 2020 | 20-03-23 | 41 | NA |
| 2020 | 20-03-24 | 8 | NA |
| 2020 | 20-03-25 | 42 | NA |
| 2020 | 20-03-25 | 39 | NA |
| 2020 | 20-03-25 | 40 | NA |
| 2020 | 20-04-10 | 22 | NA |
| 2020 | 20-04-10 | 23 | NA |
| 2020 | 20-04-10 | 45 | NA |
| 2021 | 20-12-29 | NA | 105 |
| 2021 | 21-01-03 | NA | 68 |
| 2021 | 21-01-15 | 46 | 110 |
| 2021 | 21-01-16 | 47 | 103 |
| 2021 | 21-01-17 | NA | 106 |
| 2021 | 21-01-22 | 48 | 86 |
| 2021 | 21-01-25 | 49 | 48 |
| 2021 | 21-01-26 | 50 | 72 |
| 2021 | 21-01-26 | 51 | 101 |
| 2021 | 21-01-28 | 46 | 118 |
| 2021 | 21-01-29 | 47 | 118 |
| 2021 | 21-01-29 | 52 | 94 |
| 2021 | 21-02-01 | 53 | 64 |
| 2021 | 21-02-06 | 54 | 93 |
| 2021 | 21-02-06 | 55 | 95 |
| 2021 | 21-02-07 | 56 | 115 |
| 2021 | 21-02-07 | 49 | 79 |
| 2021 | 21-02-07 | 50 | 94 |
| 2021 | 21-02-08 | 57 | 103 |
| 2021 | 21-02-10 | 46 | 127 |
| 2021 | 21-02-10 | 51 | 90 |
| 2021 | 21-02-11 | 47 | 116 |
| 2021 | 21-02-11 | 52 | 98 |
| 2021 | 21-02-16 | 48 | 100 |
| 2021 | 21-02-16 | 58 | 64 |
| 2021 | 21-02-18 | 50 | 103 |
| 2021 | 21-02-18 | 54 | 100 |
| 2021 | 21-02-19 | 59 | 113 |
| 2021 | 21-02-19 | 56 | 124 |
| 2021 | 21-02-19 | 60 | NA |
| 2021 | 21-02-19 | 61 | 103 |
| 2021 | 21-02-19 | 49 | 67 |
| 2021 | 21-02-20 | 57 | 94 |
| 2021 | 21-02-20 | 55 | 106 |
| 2021 | 21-02-21 | 62 | 97 |
| 2021 | 21-02-21 | 51 | 92 |
| 2021 | 21-02-22 | 46 | 130 |
| 2021 | 21-02-22 | 47 | 114 |
| 2021 | 21-02-22 | 52 | 108 |
| 2021 | 21-02-22 | 63 | 80 |
| 2021 | 21-02-24 | 64 | 107 |
| 2021 | 21-02-28 | 50 | 93 |
| 2021 | 21-02-28 | 48 | 105 |
| 2021 | 21-02-28 | 65 | 108 |
| 2021 | 21-03-01 | 56 | 119 |
| 2021 | 21-03-02 | 58 | 83 |
| 2021 | 21-03-02 | 66 | 68 |
| 2021 | 21-03-02 | 59 | 111 |
| 2021 | 21-03-03 | 49 | 70 |
| 2021 | 21-03-03 | 61 | 92 |
| 2021 | 21-03-03 | 67 | 106 |
| 2021 | 21-03-03 | 54 | 110 |
| 2021 | 21-03-04 | 57 | 71 |
| 2021 | 21-03-04 | 60 | 95 |
| 2021 | 21-03-04 | 55 | 120 |
| 2021 | 21-03-04 | 52 | 114 |
| 2021 | 21-03-05 | 51 | 95 |
| 2021 | 21-03-05 | 47 | 140 |
| 2021 | 21-03-06 | 46 | 135 |
| 2021 | 21-03-06 | 53 | 84 |
| 2021 | 21-03-07 | 63 | 85 |
| 2021 | 21-03-07 | 64 | 116 |
| 2021 | 21-03-09 | 62 | 112 |
| 2021 | 21-03-11 | 50 | 93 |
| 2021 | 21-03-11 | 48 | 114 |
| 2021 | 21-03-11 | 56 | 110 |
| 2021 | 21-03-13 | 66 | 101 |
| 2021 | 21-03-13 | 61 | 86 |
| 2021 | 21-03-14 | 58 | 89 |
| 2021 | 21-03-14 | 68 | 77 |
| 2021 | 21-03-14 | 57 | 71 |
| 2021 | 21-03-15 | 69 | 160 |
| 2021 | 21-03-15 | 54 | 109 |
| 2021 | 21-03-15 | 49 | 74 |
| 2021 | 21-03-16 | 47 | 133 |
| 2021 | 21-03-16 | 55 | 108 |
| 2021 | 21-03-17 | 67 | 102 |
| 2021 | 21-03-17 | 70 | 91 |
| 2021 | 21-03-18 | 60 | 169 |
| 2021 | 21-03-18 | 46 | 127 |
| 2021 | 21-03-19 | 63 | 97 |
| 2021 | 21-03-20 | 64 | 109 |
| 2021 | 21-03-20 | 62 | 111 |
| 2021 | 21-03-22 | 56 | 131 |
| 2021 | 21-03-22 | 48 | 130 |
| 2021 | 21-03-23 | 61 | 89 |
| 2021 | 21-03-23 | 50 | 93 |
| 2021 | 21-03-26 | 57 | 90 |
| 2021 | 21-03-26 | 69 | 136 |
| 2021 | 21-03-26 | 58 | 89 |
| 2021 | 21-03-26 | 54 | 115 |
| 2021 | 21-03-26 | 66 | 110 |
| 2021 | 21-03-27 | 47 | 128 |
| 2021 | 21-03-30 | 70 | 95 |
| 2021 | 21-03-30 | NA | 96 |
| 2021 | 21-03-31 | 62 | 109 |
| 2021 | 21-04-01 | 64 | 106 |
| 2021 | 21-04-02 | 48 | 110 |
| 2021 | 21-04-03 | 61 | 95 |
| 2021 | 21-04-06 | 54 | 108 |
| 2021 | 21-04-07 | 47 | 122 |
| 2021 | 21-04-07 | 58 | 80 |
| 2021 | 21-04-07 | 69 | 97 |
| 2021 | 21-04-10 | 62 | 111 |
| 2021 | 21-04-10 | 68 | 95 |
| 2021 | 21-04-10 | 71 | 99 |
| 2021 | 21-04-11 | 70 | 96 |
| 2021 | 21-04-13 | 64 | 87 |
| 2021 | 21-04-18 | 69 | 117 |
| 2021 | 21-04-18 | 47 | 103 |
| 2021 | 21-04-18 | 54 | 114 |
| 2021 | 21-04-20 | 58 | 93 |
| 2021 | 21-04-21 | 71 | 97 |
| 2021 | 21-04-24 | 70 | 95 |
| 2021 | 21-04-30 | 69 | 89 |
| 2021 | 21-05-01 | 72 | 80 |
| 2021 | 21-05-01 | 71 | 112 |
| 2021 | 21-05-06 | 70 | 107 |
| 2021 | 21-05-12 | 73 | 80 |
| 2021 | 21-05-13 | 71 | 88 |
| 2021 | 21-05-13 | 72 | 91 |
| 2021 | 21-05-20 | 70 | 102 |
| 2021 | 21-05-23 | 73 | 104 |
| 2021 | 21-05-23 | 71 | 78 |
| 2021 | 21-05-24 | 72 | 117 |
| 2021 | 21-06-04 | 73 | 101 |
| 2022 | 21-12-17 | NA | 58 |
| 2022 | 21-12-22 | NA | 118 |
| 2022 | 21-12-31 | 74 | 153 |
| 2022 | 22-01-03 | 75 | 116 |
| 2022 | 22-01-07 | 76 | 111 |
| 2022 | 22-01-09 | 77 | 97 |
| 2022 | 22-01-12 | 78 | 128 |
| 2022 | 22-01-13 | 74 | 94 |
| 2022 | 22-01-14 | 79 | 103 |
| 2022 | 22-01-15 | 80 | 89 |
| 2022 | 22-01-15 | 75 | 104 |
| 2022 | 22-01-19 | 76 | 117 |
| 2022 | 22-01-21 | 77 | 102 |
| 2022 | 22-01-22 | 81 | 103 |
| 2022 | 22-01-22 | 82 | 73 |
| 2022 | 22-01-23 | 83 | 107 |
| 2022 | 22-01-24 | 74 | 95 |
| 2022 | 22-01-26 | 75 | 118 |
| 2022 | 22-01-26 | 78 | 115 |
| 2022 | 22-01-26 | 84 | 94 |
| 2022 | 22-01-28 | 80 | 113 |
| 2022 | 22-01-29 | 79 | 102 |
| 2022 | 22-01-30 | 85 | 99 |
| 2022 | 22-02-02 | 81 | 81 |
| 2022 | 22-02-03 | 83 | 96 |
| 2022 | 22-02-03 | 77 | 91 |
| 2022 | 22-02-04 | 74 | 105 |
| 2022 | 22-02-04 | 86 | 102 |
| 2022 | 22-02-05 | 82 | 96 |
| 2022 | 22-02-06 | 75 | 141 |
| 2022 | 22-02-07 | 87 | 105 |
| 2022 | 22-02-07 | 84 | 82 |
| 2022 | 22-02-08 | 88 | 73 |
| 2022 | 22-02-08 | 78 | 122 |
| 2022 | 22-02-10 | 80 | 93 |
| 2022 | 22-02-10 | 76 | 93 |
| 2022 | 22-02-10 | 43 | 88 |
| 2022 | 22-02-10 | 89 | 96 |
| 2022 | 22-02-10 | 85 | 84 |
| 2022 | 22-02-11 | 90 | 71 |
| 2022 | 22-02-12 | 79 | 140 |
| 2022 | 22-02-13 | 81 | 111 |
| 2022 | 22-02-14 | 91 | 108 |
| 2022 | 22-02-15 | 74 | 103 |
| 2022 | 22-02-15 | 83 | 98 |
| 2022 | 22-02-16 | 77 | 104 |
| 2022 | 22-02-16 | 92 | 95 |
| 2022 | 22-02-16 | 93 | 108 |
| 2022 | 22-02-16 | 94 | 76 |
| 2022 | 22-02-17 | 86 | 122 |
| 2022 | 22-02-17 | 95 | 83 |
| 2022 | 22-02-17 | 75 | 121 |
| 2022 | 22-02-18 | 82 | 86 |
| 2022 | 22-02-18 | 84 | 84 |
| 2022 | 22-02-19 | 96 | 149 |
| 2022 | 22-02-19 | 88 | 77 |
| 2022 | 22-02-19 | 97 | 148 |
| 2022 | 22-02-20 | 80 | 100 |
| 2022 | 22-02-20 | 85 | 103 |
| 2022 | 22-02-20 | 87 | 122 |
| 2022 | 22-02-20 | 78 | 123 |
| 2022 | 22-02-20 | 76 | 60 |
| 2022 | 22-02-22 | 89 | 114 |
| 2022 | 22-02-23 | 43 | 115 |
| 2022 | 22-02-23 | 98 | 90 |
| 2022 | 22-02-23 | 79 | 143 |
| 2022 | 22-02-25 | 11 | 124 |
| 2022 | 22-02-25 | 83 | 108 |
| 2022 | 22-02-26 | 93 | 119 |
| 2022 | 22-02-26 | 91 | 115 |
| 2022 | 22-02-27 | 74 | 85 |
| 2022 | 22-02-27 | 77 | 96 |
| 2022 | 22-02-27 | 75 | 138 |
| 2022 | 22-02-28 | 95 | 94 |
| 2022 | 22-02-28 | 99 | 80 |
| 2022 | 22-03-01 | 92 | 106 |
| 2022 | 22-03-01 | 84 | 99 |
| 2022 | 22-03-01 | 96 | 119 |
| 2022 | 22-03-02 | 85 | 88 |
| 2022 | 22-03-03 | 97 | 174 |
| 2022 | 22-03-03 | 82 | 80 |
| 2022 | 22-03-03 | 100 | 101 |
| 2022 | 22-03-03 | 78 | 123 |
| 2022 | 22-03-03 | 88 | 79 |
| 2022 | 22-03-03 | 94 | 110 |
| 2022 | 22-03-04 | 76 | 106 |
| 2022 | 22-03-04 | 80 | 108 |
| 2022 | 22-03-05 | 101 | 94 |
| 2022 | 22-03-05 | 87 | 126 |
| 2022 | 22-03-06 | 81 | 129 |
| 2022 | 22-03-06 | 102 | 93 |
| 2022 | 22-03-06 | 89 | 131 |
| 2022 | 22-03-07 | 43 | 96 |
| 2022 | 22-03-08 | 103 | 106 |
| 2022 | 22-03-08 | 83 | 103 |
| 2022 | 22-03-08 | 90 | 99 |
| 2022 | 22-03-08 | 98 | 115 |
| 2022 | 22-03-09 | 11 | 139 |
| 2022 | 22-03-09 | 91 | 115 |
| 2022 | 22-03-09 | 75 | 130 |
| 2022 | 22-03-10 | 93 | 124 |
| 2022 | 22-03-10 | 104 | 58 |
| 2022 | 22-03-11 | 96 | 110 |
| 2022 | 22-03-11 | 86 | 122 |
| 2022 | 22-03-12 | 84 | 96 |
| 2022 | 22-03-12 | 85 | 109 |
| 2022 | 22-03-13 | 95 | 98 |
| 2022 | 22-03-14 | 92 | 92 |
| 2022 | 22-03-14 | 94 | 102 |
| 2022 | 22-03-14 | 100 | 76 |
| 2022 | 22-03-14 | 80 | 100 |
| 2022 | 22-03-15 | 82 | 99 |
| 2022 | 22-03-15 | 101 | 135 |
| 2022 | 22-03-15 | 78 | 120 |
| 2022 | 22-03-16 | 89 | 115 |
| 2022 | 22-03-16 | 81 | 99 |
| 2022 | 22-03-16 | 76 | 104 |
| 2022 | 22-03-17 | 105 | 89 |
| 2022 | 22-03-17 | 102 | 76 |
| 2022 | 22-03-18 | 83 | 123 |
| 2022 | 22-03-18 | 87 | 123 |
| 2022 | 22-03-18 | 43 | 104 |
| 2022 | 22-03-19 | 98 | 100 |
| 2022 | 22-03-19 | 103 | 55 |
| 2022 | 22-03-20 | 93 | 130 |
| 2022 | 22-03-20 | 75 | 121 |
| 2022 | 22-03-20 | 106 | 73 |
| 2022 | 22-03-20 | 91 | 107 |
| 2022 | 22-03-21 | 96 | 120 |
| 2022 | 22-03-21 | 11 | 132 |
| 2022 | 22-03-22 | 86 | 126 |
| 2022 | 22-03-22 | 85 | 118 |
| 2022 | 22-03-23 | 107 | 77 |
| 2022 | 22-03-24 | 104 | 114 |
| 2022 | 22-03-24 | 80 | 94 |
| 2022 | 22-03-24 | 90 | 125 |
| 2022 | 22-03-24 | 92 | 118 |
| 2022 | 22-03-24 | 100 | 96 |
| 2022 | 22-03-25 | 95 | 104 |
| 2022 | 22-03-25 | 84 | 97 |
| 2022 | 22-03-25 | 101 | 135 |
| 2022 | 22-03-25 | 94 | 59 |
| 2022 | 22-03-26 | 99 | 113 |
| 2022 | 22-03-26 | 82 | 104 |
| 2022 | 22-03-26 | 102 | 85 |
| 2022 | 22-03-26 | 108 | 55 |
| 2022 | 22-03-27 | 89 | 128 |
| 2022 | 22-03-27 | 81 | 101 |
| 2022 | 22-03-27 | 105 | 85 |
| 2022 | 22-03-28 | 83 | 104 |
| 2022 | 22-03-29 | 43 | 118 |
| 2022 | 22-03-29 | 78 | 135 |
| 2022 | 22-03-30 | 98 | 110 |
| 2022 | 22-03-30 | 103 | 90 |
| 2022 | 22-03-30 | 109 | 74 |
| 2022 | 22-03-31 | 96 | 109 |
| 2022 | 22-03-31 | 91 | 114 |
| 2022 | 22-03-31 | 93 | 127 |
| 2022 | 22-04-02 | 85 | 111 |
| 2022 | 22-04-03 | 92 | 104 |
| 2022 | 22-04-04 | 80 | 96 |
| 2022 | 22-04-04 | 95 | 96 |
| 2022 | 22-04-04 | 110 | 65 |
| 2022 | 22-04-05 | 107 | 78 |
| 2022 | 22-04-05 | 100 | 94 |
| 2022 | 22-04-05 | 101 | 124 |
| 2022 | 22-04-05 | 90 | 88 |
| 2022 | 22-04-06 | 104 | 116 |
| 2022 | 22-04-06 | 106 | 81 |
| 2022 | 22-04-06 | 99 | 102 |
| 2022 | 22-04-06 | 94 | 109 |
| 2022 | 22-04-06 | 82 | 108 |
| 2022 | 22-04-07 | 105 | 80 |
| 2022 | 22-04-07 | 102 | 76 |
| 2022 | 22-04-07 | 89 | 29 |
| 2022 | 22-04-07 | 84 | 110 |
| 2022 | 22-04-08 | 83 | 98 |
| 2022 | 22-04-08 | 108 | 74 |
| 2022 | 22-04-08 | 43 | 100 |
| 2022 | 22-04-09 | 98 | 72 |
| 2022 | 22-04-10 | 103 | 87 |
| 2022 | 22-04-11 | 91 | 122 |
| 2022 | 22-04-11 | 96 | NA |
| 2022 | 22-04-12 | 109 | 129 |
| 2022 | 22-04-14 | 92 | 104 |
| 2022 | 22-04-15 | 101 | 122 |
| 2022 | 22-04-16 | 100 | 113 |
| 2022 | 22-04-16 | 90 | 97 |
| 2022 | 22-04-17 | 106 | 90 |
| 2022 | 22-04-17 | 95 | 94 |
| 2022 | 22-04-17 | 102 | 81 |
| 2022 | 22-04-17 | 107 | 90 |
| 2022 | 22-04-17 | 99 | 99 |
| 2022 | 22-04-18 | 105 | 73 |
| 2022 | 22-04-19 | 43 | 89 |
| 2022 | 22-04-19 | 82 | 124 |
| 2022 | 22-04-19 | 104 | 121 |
| 2022 | 22-04-20 | 110 | 51 |
| 2022 | 22-04-20 | 111 | 136 |
| 2022 | 22-04-22 | 91 | 126 |
| 2022 | 22-04-23 | 92 | 105 |
| 2022 | 22-04-24 | 109 | 112 |
| 2022 | 22-04-25 | 101 | 135 |
| 2022 | 22-04-27 | 90 | 98 |
| 2022 | 22-04-27 | 107 | 88 |
| 2022 | 22-04-27 | 100 | 107 |
| 2022 | 22-04-29 | 106 | 81 |
| 2022 | 22-04-30 | 43 | 86 |
| 2022 | 22-05-03 | 111 | 130 |
| 2022 | 22-05-07 | 107 | NA |
| 2022 | 22-05-08 | 100 | NA |
| 2022 | 22-05-08 | 90 | NA |
| 2022 | 22-05-08 | 109 | NA |
| 2022 | 22-05-11 | 43 | NA |
| 2022 | 22-05-14 | 111 | NA |
| 2022 | 22-05-19 | 100 | NA |
| 2022 | 22-05-20 | 107 | NA |
| 2022 | 22-05-20 | 90 | NA |
| 2022 | 22-05-20 | 112 | NA |
| 2022 | 22-05-25 | 111 | NA |
| 2022 | 22-06-05 | 111 | NA |
| 2022 | 22-06-18 | NA | NA |
| 2022 | 22-06-19 | NA | NA |
| 2022 | 22-07-04 | NA | NA |
| 2023 | 22-12-29 | NA | 163 |
| 2023 | 23-01-11 | 113 | 67 |
| 2023 | 23-01-12 | 1 | 170 |
| 2023 | 23-01-16 | 114 | 70 |
| 2023 | 23-01-17 | 115 | 90 |
| 2023 | 23-01-21 | 39 | 142 |
| 2023 | 23-01-23 | 116 | 57 |
| 2023 | 23-01-24 | 1 | 164 |
| 2023 | 23-01-25 | 117 | 110 |
| 2023 | 23-01-25 | 118 | 133 |
| 2023 | 23-01-26 | 113 | 81 |
| 2023 | 23-01-28 | 119 | 86 |
| 2023 | 23-01-28 | 120 | 121 |
| 2023 | 23-01-29 | 121 | 85 |
| 2023 | 23-01-30 | 114 | 79 |
| 2023 | 23-01-30 | 122 | 64 |
| 2023 | 23-01-31 | 123 | 120 |
| 2023 | 23-02-01 | 115 | 96 |
| 2023 | 23-02-01 | 124 | 109 |
| 2023 | 23-02-01 | 125 | 148 |
| 2023 | 23-02-02 | 126 | 58 |
| 2023 | 23-02-02 | 39 | 116 |
| 2023 | 23-02-03 | 127 | 54 |
| 2023 | 23-02-03 | 128 | 96 |
| 2023 | 23-02-03 | 116 | 74 |
| 2023 | 23-02-05 | 129 | 81 |
| 2023 | 23-02-06 | 130 | 97 |
| 2023 | 23-02-06 | 131 | 121 |
| 2023 | 23-02-06 | 21 | 94 |
| 2023 | 23-02-06 | 1 | 153 |
| 2023 | 23-02-07 | 132 | 62 |
| 2023 | 23-02-07 | 117 | 103 |
| 2023 | 23-02-07 | 113 | 79 |
| 2023 | 23-02-09 | 119 | 63 |
| 2023 | 23-02-09 | 133 | 115 |
| 2023 | 23-02-09 | 134 | 62 |
| 2023 | 23-02-09 | 120 | 107 |
| 2023 | 23-02-10 | 135 | 106 |
| 2023 | 23-02-10 | 121 | 75 |
| 2023 | 23-02-10 | 118 | 129 |
| 2023 | 23-02-11 | 136 | 69 |
| 2023 | 23-02-11 | 114 | 93 |
| 2023 | 23-02-11 | 123 | 127 |
| 2023 | 23-02-12 | 124 | 101 |
| 2023 | 23-02-13 | 126 | 75 |
| 2023 | 23-02-14 | 115 | 111 |
| 2023 | 23-02-15 | 116 | 81 |
| 2023 | 23-02-15 | 137 | 40 |
| 2023 | 23-02-15 | 128 | 89 |
| 2023 | 23-02-15 | 125 | 125 |
| 2023 | 23-02-15 | 39 | 109 |
| 2023 | 23-02-15 | 127 | 66 |
| 2023 | 23-02-16 | 129 | 72 |
| 2023 | 23-02-17 | 1 | 150 |
| 2023 | 23-02-18 | 131 | 130 |
| 2023 | 23-02-18 | 21 | 91 |
| 2023 | 23-02-18 | 130 | 111 |
| 2023 | 23-02-19 | 117 | NA |
| 2023 | 23-02-19 | 132 | 84 |
| 2023 | 23-02-19 | 35 | 96 |
| 2023 | 23-02-19 | 113 | 90 |
| 2023 | 23-02-20 | 119 | 87 |
| 2023 | 23-02-20 | 120 | 105 |
| 2023 | 23-02-20 | 138 | 99 |
| 2023 | 23-02-21 | 139 | 27 |
| 2023 | 23-02-21 | 140 | 114 |
| 2023 | 23-02-21 | 133 | 113 |
| 2023 | 23-02-21 | 135 | 79 |
| 2023 | 23-02-22 | 141 | 126 |
| 2023 | 23-02-23 | 136 | 87 |
| 2023 | 23-02-23 | 134 | 65 |
| 2023 | 23-02-23 | 123 | 139 |
| 2023 | 23-02-23 | 124 | 101 |
| 2023 | 23-02-23 | 118 | 134 |
| 2023 | 23-02-23 | 114 | 90 |
| 2023 | 23-02-24 | 126 | 50 |
| 2023 | 23-02-25 | 121 | 91 |
| 2023 | 23-02-26 | 129 | 58 |
| 2023 | 23-02-26 | 116 | 36 |
| 2023 | 23-02-26 | 125 | 131 |
| 2023 | 23-02-26 | 127 | 68 |
| 2023 | 23-02-27 | 128 | 102 |
| 2023 | 23-02-27 | 39 | 75 |
| 2023 | 23-02-27 | 115 | 106 |
| 2023 | 23-02-28 | 142 | 112 |
| 2023 | 23-02-28 | 1 | 152 |
| 2023 | 23-02-28 | 143 | 96 |
| 2023 | 23-03-01 | 131 | 140 |
| 2023 | 23-03-01 | 21 | 97 |
| 2023 | 23-03-01 | 144 | 87 |
| 2023 | 23-03-02 | 113 | 97 |
| 2023 | 23-03-02 | 145 | 104 |
| 2023 | 23-03-02 | 132 | 88 |
| 2023 | 23-03-02 | 117 | 114 |
| 2023 | 23-03-02 | 137 | 24 |
| 2023 | 23-03-03 | 119 | 96 |
| 2023 | 23-03-03 | 35 | 89 |
| 2023 | 23-03-03 | 146 | 67 |
| 2023 | 23-03-04 | 130 | 92 |
| 2023 | 23-03-04 | 139 | 83 |
| 2023 | 23-03-04 | 138 | 105 |
| 2023 | 23-03-04 | 140 | 90 |
| 2023 | 23-03-05 | 120 | 113 |
| 2023 | 23-03-05 | 133 | 101 |
| 2023 | 23-03-05 | 135 | 100 |
| 2023 | 23-03-05 | 147 | 81 |
| 2023 | 23-03-06 | 123 | 152 |
| 2023 | 23-03-06 | 114 | 98 |
| 2023 | 23-03-06 | 124 | 97 |
| 2023 | 23-03-06 | 148 | 95 |
| 2023 | 23-03-07 | 126 | 108 |
| 2023 | 23-03-07 | 118 | 149 |
| 2023 | 23-03-07 | 134 | 80 |
| 2023 | 23-03-08 | 141 | 77 |
| 2023 | 23-03-08 | 121 | 82 |
| 2023 | 23-03-08 | 149 | 87 |
| 2023 | 23-03-09 | 125 | 130 |
| 2023 | 23-03-09 | 128 | 103 |
| 2023 | 23-03-09 | 127 | 77 |
| 2023 | 23-03-09 | 129 | 92 |
| 2023 | 23-03-09 | 116 | 40 |
| 2023 | 23-03-09 | 150 | 44 |
| 2023 | 23-03-10 | 151 | 137 |
| 2023 | 23-03-11 | 1 | 156 |
| 2023 | 23-03-11 | 152 | 101 |
| 2023 | 23-03-11 | 39 | 112 |
| 2023 | 23-03-11 | 153 | 73 |
| 2023 | 23-03-11 | 142 | 126 |
| 2023 | 23-03-11 | 115 | 104 |
| 2023 | 23-03-12 | 154 | 66 |
| 2023 | 23-03-12 | 131 | 133 |
| 2023 | 23-03-13 | 137 | 50 |
| 2023 | 23-03-13 | 143 | 100 |
| 2023 | 23-03-13 | 145 | 95 |
| 2023 | 23-03-13 | 113 | 86 |
| 2023 | 23-03-13 | 21 | 102 |
| 2023 | 23-03-13 | 132 | 90 |
| 2023 | 23-03-13 | 117 | NA |
| 2023 | 23-03-14 | 130 | 90 |
| 2023 | 23-03-15 | 146 | 79 |
| 2023 | 23-03-15 | 119 | 118 |
| 2023 | 23-03-15 | 35 | 98 |
| 2023 | 23-03-16 | 138 | 101 |
| 2023 | 23-03-16 | 147 | 101 |
| 2023 | 23-03-16 | 139 | 75 |
| 2023 | 23-03-16 | 140 | 83 |
| 2023 | 23-03-16 | 133 | 98 |
| 2023 | 23-03-17 | 135 | 103 |
| 2023 | 23-03-17 | 126 | 107 |
| 2023 | 23-03-17 | 124 | NA |
| 2023 | 23-03-18 | 123 | 130 |
| 2023 | 23-03-18 | 134 | 70 |
| 2023 | 23-03-18 | 155 | 88 |
| 2023 | 23-03-19 | 149 | 123 |
| 2023 | 23-03-19 | 118 | 125 |
| 2023 | 23-03-19 | 121 | 87 |
| 2023 | 23-03-19 | 128 | 104 |
| 2023 | 23-03-19 | 129 | 88 |
| 2023 | 23-03-19 | 114 | NA |
| 2023 | 23-03-19 | 141 | 100 |
| 2023 | 23-03-21 | 151 | 142 |
| 2023 | 23-03-21 | 127 | 90 |
| 2023 | 23-03-22 | 1 | 153 |
| 2023 | 23-03-22 | 153 | 84 |
| 2023 | 23-03-22 | 125 | 87 |
| 2023 | 23-03-22 | 142 | 96 |
| 2023 | 23-03-22 | 152 | 103 |
| 2023 | 23-03-22 | 148 | 113 |
| 2023 | 23-03-23 | 39 | 97 |
| 2023 | 23-03-23 | 154 | 107 |
| 2023 | 23-03-23 | 137 | 71 |
| 2023 | 23-03-23 | 150 | 90 |
| 2023 | 23-03-24 | 131 | 135 |
| 2023 | 23-03-24 | 143 | 119 |
| 2023 | 23-03-24 | 21 | NA |
| 2023 | 23-03-24 | 113 | 91 |
| 2023 | 23-03-24 | 115 | 114 |
| 2023 | 23-03-24 | 132 | 104 |
| 2023 | 23-03-25 | 145 | 121 |
| 2023 | 23-03-25 | 119 | 119 |
| 2023 | 23-03-26 | 35 | 95 |
| 2023 | 23-03-26 | 156 | 38 |
| 2023 | 23-03-26 | 146 | 83 |
| 2023 | 23-03-26 | 147 | 99 |
| 2023 | 23-03-26 | 133 | 85 |
| 2023 | 23-03-27 | 139 | 83 |
| 2023 | 23-03-27 | 124 | 116 |
| 2023 | 23-03-27 | 135 | 101 |
| 2023 | 23-03-28 | 138 | 114 |
| 2023 | 23-03-28 | 157 | 57 |
| 2023 | 23-03-29 | 123 | 163 |
| 2023 | 23-03-29 | 128 | 113 |
| 2023 | 23-03-29 | 134 | 81 |
| 2023 | 23-03-30 | 121 | 80 |
| 2023 | 23-03-30 | 118 | 122 |
| 2023 | 23-03-30 | 129 | 72 |
| 2023 | 23-03-30 | 158 | 98 |
| 2023 | 23-03-30 | 149 | 107 |
| 2023 | 23-03-30 | 114 | 68 |
| 2023 | 23-03-31 | 159 | 99 |
| 2023 | 23-04-01 | 153 | 88 |
| 2023 | 23-04-01 | 151 | 135 |
| 2023 | 23-04-01 | 152 | 100 |
| 2023 | 23-04-01 | 148 | 117 |
| 2023 | 23-04-02 | 137 | 86 |
| 2023 | 23-04-02 | 125 | 148 |
| 2023 | 23-04-02 | 39 | 96 |
| 2023 | 23-04-03 | 127 | 77 |
| 2023 | 23-04-04 | 131 | 125 |
| 2023 | 23-04-04 | 132 | 105 |
| 2023 | 23-04-04 | 142 | 112 |
| 2023 | 23-04-04 | 119 | 137 |
| 2023 | 23-04-05 | 154 | 95 |
| 2023 | 23-04-05 | 21 | 102 |
| 2023 | 23-04-05 | 133 | 89 |
| 2023 | 23-04-05 | 145 | 113 |
| 2023 | 23-04-05 | 147 | NA |
| 2023 | 23-04-05 | 160 | 93 |
| 2023 | 23-04-05 | 161 | 104 |
| 2023 | 23-04-06 | 146 | 56 |
| 2023 | 23-04-06 | 35 | 94 |
| 2023 | 23-04-06 | 124 | 109 |
| 2023 | 23-04-06 | 156 | NA |
| 2023 | 23-04-07 | 157 | 111 |
| 2023 | 23-04-07 | 135 | NA |
| 2023 | 23-04-07 | 162 | 98 |
| 2023 | 23-04-08 | 138 | 117 |
| 2023 | 23-04-09 | 123 | 113 |
| 2023 | 23-04-09 | 163 | 94 |
| 2023 | 23-04-09 | 164 | 99 |
| 2023 | 23-04-10 | 158 | 109 |
| 2023 | 23-04-10 | 141 | 104 |
| 2023 | 23-04-10 | 159 | 76 |
| 2023 | 23-04-10 | 118 | 112 |
| 2023 | 23-04-10 | 149 | 89 |
| 2023 | 23-04-10 | 165 | 84 |
| 2023 | 23-04-11 | 152 | 79 |
| 2023 | 23-04-11 | 134 | 88 |
| 2023 | 23-04-11 | 153 | 95 |
| 2023 | 23-04-11 | 148 | 114 |
| 2023 | 23-04-12 | 137 | 83 |
| 2023 | 23-04-12 | 151 | 161 |
| 2023 | 23-04-13 | 125 | 92 |
| 2023 | 23-04-13 | 39 | 83 |
| 2023 | 23-04-14 | 119 | 121 |
| 2023 | 23-04-14 | 154 | 113 |
| 2023 | 23-04-15 | 132 | 111 |
| 2023 | 23-04-15 | 147 | 88 |
| 2023 | 23-04-15 | 145 | 116 |
| 2023 | 23-04-15 | 133 | 101 |
| 2023 | 23-04-16 | 142 | 97 |
| 2023 | 23-04-17 | 35 | 108 |
| 2023 | 23-04-17 | 162 | 106 |
| 2023 | 23-04-18 | 138 | 112 |
| 2023 | 23-04-18 | 161 | 89 |
| 2023 | 23-04-18 | 135 | 87 |
| 2023 | 23-04-19 | 160 | 99 |
| 2023 | 23-04-20 | 157 | 84 |
| 2023 | 23-04-20 | 141 | 71 |
| 2023 | 23-04-20 | 159 | 94 |
| 2023 | 23-04-22 | 148 | 91 |
| 2023 | 23-04-22 | 153 | 52 |
| 2023 | 23-04-22 | 158 | 106 |
| 2023 | 23-04-22 | 151 | 137 |
| 2023 | 23-04-23 | 166 | NA |
| 2023 | 23-04-24 | 167 | 77 |
| 2023 | 23-04-24 | 154 | 71 |
| 2023 | 23-04-25 | 119 | 108 |
| 2023 | 23-04-26 | 125 | 100 |
| 2023 | 23-04-26 | 133 | 87 |
| 2023 | 23-04-26 | 132 | 98 |
| 2023 | 23-04-27 | 145 | NA |
| 2023 | 23-04-27 | 161 | 108 |
| 2023 | 23-04-29 | 138 | 114 |
| 2023 | 23-04-29 | 162 | 71 |
| 2023 | 23-05-01 | 141 | NA |
| 2023 | 23-05-01 | 159 | NA |
| 2023 | 23-05-03 | 158 | NA |
| 2023 | 23-05-03 | 148 | NA |
| 2023 | 23-05-04 | 151 | NA |
| 2023 | 23-05-04 | 154 | NA |
| 2023 | 23-05-07 | 166 | NA |
| 2023 | 23-05-07 | 167 | NA |
| 2023 | 23-05-07 | 145 | NA |
| 2023 | 23-05-07 | 161 | NA |
| 2023 | 23-05-07 | 133 | NA |
| 2023 | 23-05-08 | 132 | NA |
| 2023 | 23-05-10 | 138 | NA |
| 2023 | 23-05-14 | 158 | NA |
| 2023 | 23-05-15 | 148 | NA |
| 2023 | 23-05-16 | 161 | NA |
| 2023 | 23-05-18 | 145 | NA |
| 2023 | 23-05-19 | 167 | NA |
| 2023 | NA | NA | 87 |
