# Supplementary material for: Power analyses to inform clutch sampling design to determine the breeding sex ratio in populations with multiple paternity
Source: PeerJ. 2025 Oct 28;13:e20165. doi: 10.7717/peerj.20165 (PMC12577575; doi:10.7717/peerj.20165)
Supplement: Supplemental Information 2 — Values in each cell are the average proportion of green turtle clutches that had eggs fertilized by the F th father in clutches analyzed genetically. No study showed any wild green turtle clutches that had eggs fertilized by more than five fathers. [file peerj-13-20165-s002.docx]

| **Source** | **Location** | **Clutches sampled** | **Average offspring** **per clutch sampled** | ***F* = 1** | ***F* = 2** | ***F* = 3** | ***F* = 4** | ***F* = 5** | **Total** |
| --- | --- | --- | --- | --- | --- | --- | --- | --- | --- |
| (Alfaro-Núñez, Jensen & Abreu-Grobois, 2015) | Costa Rica | 12.000 | 45 | 0.083 | 0.333 | 0.333 | 0.083 | 0.167 | **1.000** |
| (Chassin-Noria et al., 2017) | Mexico | 16.000 | 23 | 0.250 | 0.250 | 0.313 | 0.188 | 0.000 | **1.000** |
| (Ekanayake et al., 2013) | Sri Lanka | 24.000 | 10 | 0.375 | 0.542 | 0.083 | 0.000 | 0.000 | **1.000** |
| (Fitzsimmons, 1998) | Southern Great Barrier Reef | 22.000 | 41 | 0.909 | 0.091 | 0.000 | 0.000 | 0.000 | **1.000** |
| (Lee, Hays & Avise, 2004) | Ascension Island | 17.000 | 39 | 0.412 | 0.294 | 0.235 | 0.000 | 0.059 | **1.000** |
| (Purnama, Zamani & Farajallah, 2013) | Indonesia | 10.000 | 12 | 0.500 | 0.500 | 0.000 | 0.000 | 0.000 | **1.000** |
| (Türkozan et al., 2019) | Turkey | 22.000 | 15 | 0.409 | 0.364 | 0.227 | 0.000 | 0.000 | **1.000** |
| (Wright et al., 2012) | Cyprus | 94.000 | 22 | 0.766 | 0.170 | 0.064 | 0.000 | 0.000 | **1.000** |
| **Average proportion** | | | | **0.463** | **0.318** | **0.157** | **0.034** | **0.028** | **1.000** |
