# Supplementary material for: Power analyses to inform clutch sampling design to determine the breeding sex ratio in populations with multiple paternity
Source: PeerJ. 2025 Oct 28;13:e20165. doi: 10.7717/peerj.20165 (PMC12577575; doi:10.7717/peerj.20165)
Supplement: Supplemental Information 3 — Some rows do not appear to add up to 1.000 due to rounding. [file peerj-13-20165-s003.docx]

| Paternal contribution mode | Probability of F fathers identified given a sample size of 32 offspring | | | | | Fathers actual | Probability of F fathers identified given a sample size of 96 offspring | | | | |
| --- | --- | --- | --- | --- | --- | --- | --- | --- | --- | --- | --- |
|  | F = 1 | F = 2 | F = 3 | F = 4 | F = 5 |  | F = 1 | F = 2 | F = 3 | F = 4 | F = 5 |
| Random | 1 | - | - | - | - | 1 | 1 | - | - | - | - |
|  | 0 | 1 | - | - | - | 2 | 0 | 1 | - | - | - |
|  | 0 | 0 | 1 | - | - | 3 | 0 | 0 | 1 | - | - |
|  | 0 | 0 | 0.001 | 1.000 | - | 4 | 0 | 0 | 0 | 1 | - |
|  | 0 | 0 | 0 | 0.004 | 0.996 | 5 | 0 | 0 | 0 | 0 | 1 |
| Exponential | 1 | - | - | - | - | 1 | 1 | - | - | - | - |
|  | 0 | 1 | - | - | - | 2 | 0 | 1 | - | - | - |
|  | 0 | 0 | 1 | - | - | 3 | 0 | 0 | 1 | - | - |
|  | 0 | 0 | 0.027 | 0.973 | - | 4 | 0 | 0 | 0 | 1 | - |
|  | 0 | 0 | 0.016 | 0.233 | 0.750 | 5 | 0 | 0 | 0 | 0.010 | 0.990 |
| Dominant 50 | 1 | - | - | - | - | 1 | 1 | - | - | - | - |
|  | 0 | 1 | - | - | - | 2 | 0 | 1 | - | - | - |
|  | 0 | 0 | 1 | - | - | 3 | 0 | 0 | 1 | - | - |
|  | 0 | 0 | 0.009 | 0.992 | - | 4 | 0 | 0 | 0 | 1 | - |
|  | 0 | 0 | 0.001 | 0.054 | 0.945 | 5 | 0 | 0 | 0 | 0.001 | 0.999 |
| Dominant 70 | 1 | - | - | - | - | 1 | 1 | - | - | - | - |
|  | 0 | 1 | - | - | - | 2 | 0 | 1 | - | - | - |
|  | 0 | 0.011 | 0.989 | - | - | 3 | 0 | 0 | 1 | - | - |
|  | 0 | 0.002 | 0.099 | 0.898 | - | 4 | 0 | 0 | 0.001 | 0.999 | - |
|  | 0 | 0.001 | 0.030 | 0.268 | 0.701 | 5 | 0 | 0 | 0 | 0.008 | 0.992 |
| Dominant 90 | 1 | - | - | - | - | 1 | 1 | - | - | - | - |
|  | 0.033 | 0.967 | - | - | - | 2 | 0.001 | 1.000 | - | - | - |
|  | 0.035 | 0.318 | 0.647 | - | - | 3 | 0.001 | 0.028 | 0.972 | - | - |
|  | 0.034 | 0.227 | 0.456 | 0.283 | - | 4 | 0.001 | 0.010 | 0.142 | 0.847 | - |
|  | 0.034 | 0.194 | 0.378 | 0.306 | 0.088 | 5 | 0.001 | 0.007 | 0.061 | 0.302 | 0.630 |
| Mixed Dominant | 1 | - | - | - | - | 1 | 1 | - | - | - | - |
|  | 0.012 | 0.988 | - | - | - | 2 | 0 | 1 | - | - | - |
|  | 0.012 | 0.110 | 0.878 | - | - | 3 | 0 | 0.009 | 0.991 | - | - |
|  | 0.012 | 0.076 | 0.189 | 0.723 | - | 4 | 0 | 0.003 | 0.049 | 0.947 | - |
|  | 0.012 | 0.065 | 0.138 | 0.209 | 0.575 | 5 | 0 | 0.002 | 0.020 | 0.105 | 0.872 |
