# Supplementary material for: Power analyses to inform clutch sampling design to determine the breeding sex ratio in populations with multiple paternity
Source: PeerJ. 2025 Oct 28;13:e20165. doi: 10.7717/peerj.20165 (PMC12577575; doi:10.7717/peerj.20165)
Supplement: Supplemental Information 4 — Color indicates the upper edge of the confidence band, with solid fill representing simulations with the Random paternal contribution mode and lines representing simulations with the Dominant 90 paternal contribution mode. Within each panel, the x-axis represents the proportion of clutches across the entire nesting season that were sampled, and the y-axis represents the operational sex ratio. Columns of panels show different distributions of polyandry, with results from simulations with decreasing probabilities (“Decreasing polyandry” in panels (A), (B), and (C)) and uniform probabilities (“Uniform polyandry, in panels (D), (E), (F)) of females breeding with one to five males. Rows of panels show different distributions of polygyny, with results from simulations with decreasing probabilities of mating with 1–5 females (“Decreasing polygyny”, panels (A), (D)), uniform probabilities of mating with 1–5 females (“Uniform polygyny”, panels (B), (E)), and zero probabilities of males breeding with more than one female (“No polygyny”, panels (C), (F)). [file peerj-13-20165-s004.pdf]

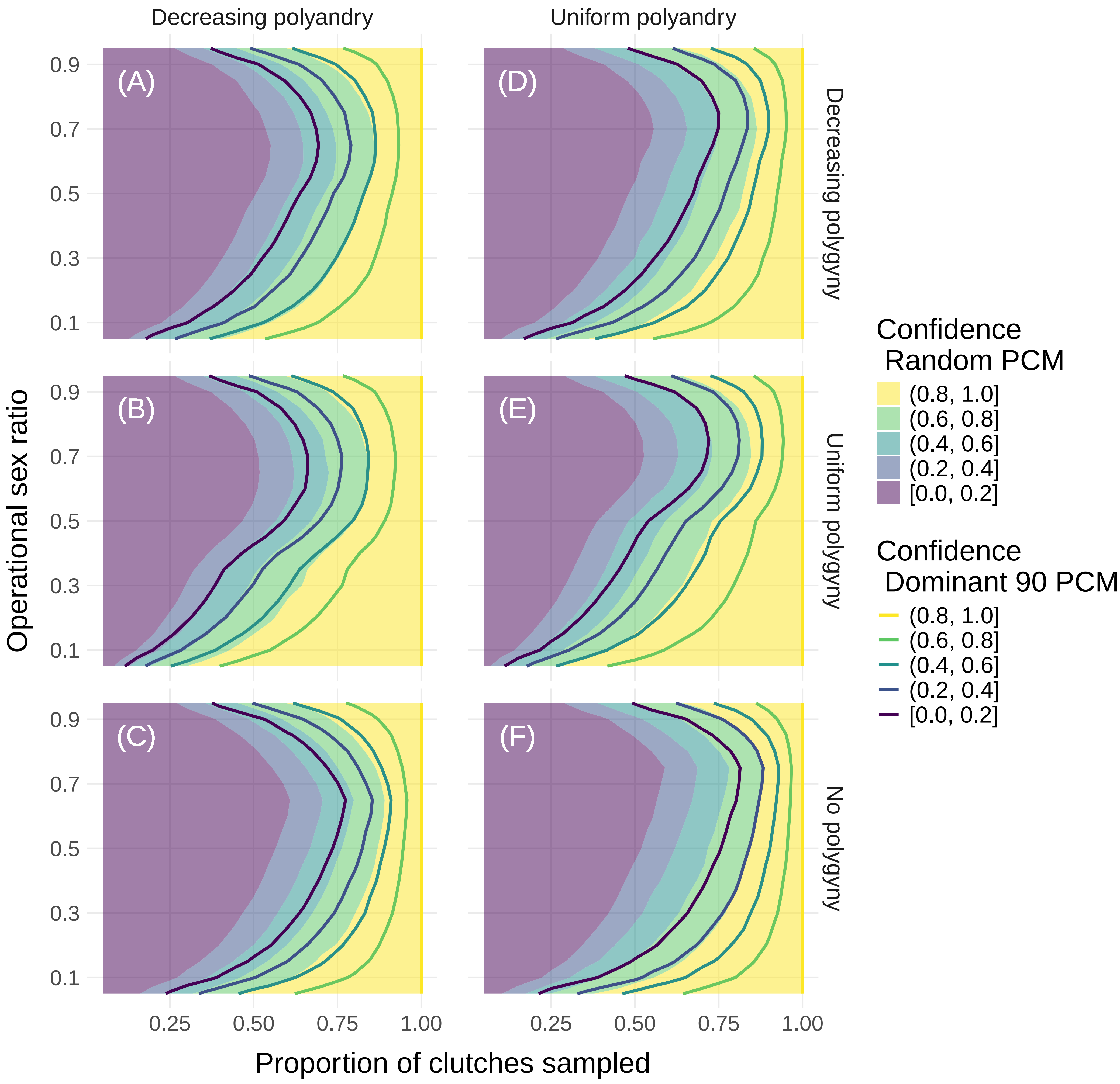

Decreasing polyandry
Uniform polyandry
(A)
(B)
(C)
(D)
(E)
(F)
Decreasing polygyny
Uniform polygyny
No polygyny
0.9
0.7
0.5
0.3
0.1
0.25
0.50
0.75
1.00
Operational sex ratio
Proportion of clutches sampled
Confidence Random PCM
(0.8, 1.0]
(0.6, 0.8]
(0.4, 0.6]
(0.2, 0.4]
[0.0, 0.2]
Confidence Dominant 90 PCM
(0.8, 1.0]
(0.6, 0.8]
(0.4, 0.6]
(0.2, 0.4]
[0.0, 0.2]
