# Supplementary material for: Power analyses to inform clutch sampling design to determine the breeding sex ratio in populations with multiple paternity
Source: PeerJ. 2025 Oct 28;13:e20165. doi: 10.7717/peerj.20165 (PMC12577575; doi:10.7717/peerj.20165)
Supplement: Supplemental Information 5 — Color indicates the proportion of simulations with offspring sample sizes of 32 where the minimum required fathers were correctly identified, with solid fill representing simulations with the Random paternal contribution mode and lines representing simulations with the Dominant 90 paternal contribution mode. Within each panel, the x-axis represents the proportion of clutches across the entire nesting season that were sampled, and the y-axis represents the operational sex ratio. Columns of panels show different minimum proportions of fathers to ID to be counted as a “success”: 90% of all fathers (panels (A), (B), (C), (D) and 100% of fathers (panels (E), (F), (G), (H)). Rows of panels show different starting population sizes of adults available for breeding: 100 (panels (A), (E)), 200 (panels (B), (F)), 500 (panels (C), (G)), and 1,000 (panels (D), (H)). The mating system modeled had uniform probabilities of mating with one to five mates (“uniform polyandry” and “uniform polygyny”). [file peerj-13-20165-s005.pdf]

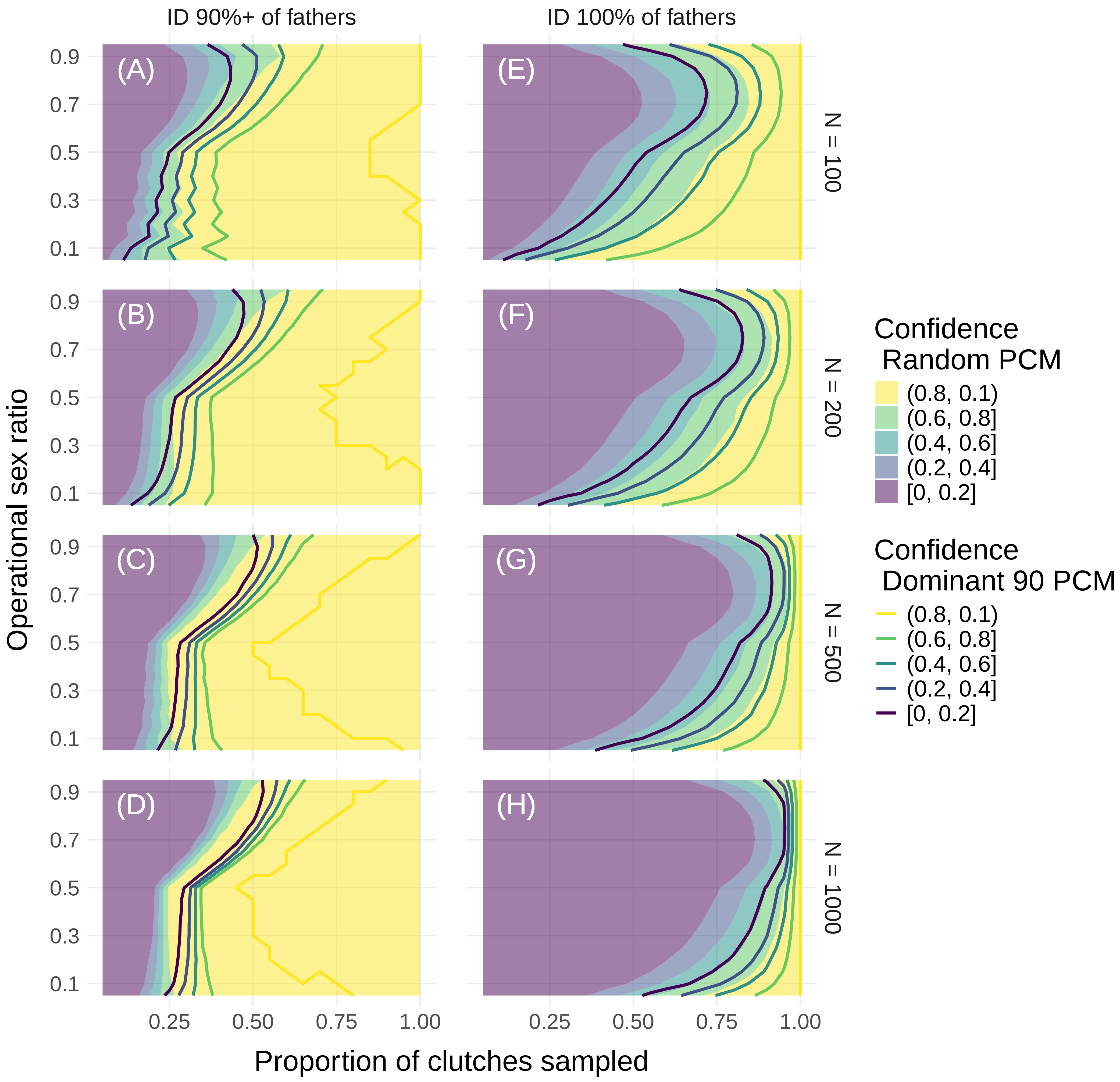

ID 90%+ of fathers
ID 100% of fathers
(A)
(B)
(C)
(D)
(E)
(F)
(G)
(H)
N = 100
N = 200
N = 500
N = 1000
Operational sex ratio
0.9
0.7
0.5
0.3
0.1
0.25
0.50
0.75
1.00
Proportion of clutches sampled
Confidence Random PCM
(0.8, 0.1)
(0.6, 0.8]
(0.4, 0.6]
(0.2, 0.4]
[0, 0.2]
Confidence Dominant 90 PCM
(0.8, 0.1)
(0.6, 0.8]
(0.4, 0.6]
(0.2, 0.4]
[0, 0.2]
